# Transfer Validity of Pediatric Supracondylar Humeral Fracture Pin Placement Practice on In-Theater Performance by Orthopedic Trainees Using an Augmented Reality Simulator: Protocol for a Pilot Interventional Cohort Study With a Retrospective Comparator Cohort

**DOI:** 10.2196/38282

**Published:** 2023-08-02

**Authors:** Joyce Guo, Phil Blyth, Kari Clifford, Nikki Hooper, Haemish Crawford

**Affiliations:** 1 Department of the Dean Otago Medical School University of Otago Dunedin New Zealand; 2 Surgical Outcomes Research Centre Otago, Department of Surgical Sciences Otago Medical School University of Otago Dunedin New Zealand; 3 Department of Orthopaedic Surgery Otago Medical School University of Otago Christchurch New Zealand; 4 Department of Orthopaedics Starship Children's Hospital Auckland New Zealand

**Keywords:** pediatric orthopedics, augmented reality simulator, supracondylar humeral fractures, closed reduction and percutaneous pinning, transfer validity, fracture, surgeons, education, practice, trainees, pediatric, orthopedic, training, surgical procedure

## Abstract

**Background:**

Supracondylar humeral fractures (SCHF) are a common cause of orthopedic morbidity in pediatric populations across the world. The treatment of this fracture is likely one of the first procedures involving x-ray–guided wire insertion that trainee orthopedic surgeons will encounter in their career. Traditional surgical training methods of “see one, do one, teach one” are reliant on the presence of real-world cases and must be conducted within an operative environment. We have developed an augmented reality simulator that allows trainees to practice this procedure in a radiation-free environment at no extra risk to patients.

**Objective:**

This study aims to examine whether training on a simulator in addition to traditional surgical training improves the in-theater performance of trainees.

**Methods:**

This multicenter, interventional cohort study will involve orthopedic trainees from New Zealand in their first year of advanced training between 2019 and 2023. Advanced trainees with no simulator exposure who were in their first year in 2019-2021 will form the comparator cohort, while those in the years 2022-2023 will receive additional regular simulator training as the intervention cohort. The comparator cohort’s performance in pediatric SCHF surgery will be retrospectively audited using routinely collected operative outcomes and parameters over a 6-month period. Data on the performance of the intervention cohorts will be collected in the same way over a comparable period. The data collected for both groups will be used to determine whether additional training with an augmented reality training shows improved real-world surgical outcomes compared to traditional surgical training.

**Results:**

As of February 2022, a total of 8 retrospective comparator trainees have been recruited by email. The study is financially supported through an external grant from the Wishbone Orthopaedic Research Foundation of New Zealand (September 2021) and an internal research grant from the University of Otago (July 2021).

**Conclusions:**

This protocol has been approved by the University of Otago Health Ethics committee (reference HD21/087), and the study is due for completion in 2024. This protocol may assist other researchers conducting similar studies in the field.

**Trial Registration:**

Australian New Zealand Clinical Trials Registry ACTRN12623000816651; https://tinyurl.com/mtdkecwb

**International Registered Report Identifier (IRRID):**

DERR1-10.2196/38282

## Introduction

### Background

Supracondylar humeral fractures (SCHF) of the elbow are one of the most common orthopedic causes of morbidity in children. SCHF represent 3.3% to 16.6% of all childhood fractures, 32.6% of upper extremity fractures, and 57.5% of elbow fractures across localities [1-4]. The Gartland classification system is used for characterizing the extension type of SCHF and is dependent on the degree of humeral fragment displacement [5]. The gold standard for the management of operative cases is closed reduction and percutaneous pinning (CRPP), unless severe neurovascular compromise or an open fracture is present [6-8]. CRPP is seen as a more effective and safer method for treating displaced SCHF than nonoperative and open reduction methods [9-12].

### Current Training

The status quo of apprenticeship-style orthopedic training does not enable standardized hands-on skill practice outside of the operating room. The “see one, do one, teach one” methodology is becoming increasingly insufficient as trainees face a decrease in work hours for necessary well-being needs. This decrease in work hours means there are fewer opportunities for trainees to participate in and therefore learn from real cases. Limiting training to the operating room also incurs both financial and opportunity costs to the health care system as trainees require more time to complete procedures than consultants [13,14]. In addition to costs and reduced availability of the current method of training, there is also the question of whether apprenticeship-style teaching is really the most effective form of teaching available. Numerous studies on learning and teaching for adults have shown that independent, deliberate, and repeated practice is most effective for skill acquisition [15-18]. By nature, apprenticeship-style training allows for the repetition of individual case types but not a deliberate practice of certain aspects of the skills, such as only percutaneous pinning in CRPP.

### An Alternative Training Method

Improvements in computer technology have afforded the development of a new method of surgical training—augmented reality (AR) simulation. Using AR technology, skill acquisition can take place before entering the operating room. CRPP requires a high level of hand-eye coordination, visuospatial reasoning to interpret the X-ray image, and haptic interpretation to be able to feel the wire entering or leaving the bone. One such example was developed by our research team. The “BoneDoc AR” simulator (University of Otago) that we have produced uses an iPad app to provide real-time simulated X-ray images, while trainees are drilling through a physical model of the elbow. This physical model is made of silicone soft tissue and 3D-printed plastic bones. In addition, the simulator can provide objective measures of proficiency, such as exact pin placement, which are not possible without the use of computed tomography scans. These objective measures may be of value in providing feedback on skills acquisition during surgical training. 

### Study Overview

Appropriate validation of the BoneDoc AR simulator is necessary to determine its usefulness in surgical training. Transfer validity, the ultimate test of a surgical simulator, is the transfer of skills acquired on a simulator into the real-world operating theater. Validity tests are necessary to ensure the efficacy of a simulator in a training environment [19]. There are few surgical AR simulators, which have shown positive transfer validity, and none on AR simulators of SCHF treatment [20]. There is a clear paucity in the literature of this field, and there is a need for further research. This pilot study aims to examine the transfer validity of orthopedic trainee practice on the BoneDoc AR simulator.

We hypothesize that training with the simulator will improve in-theater performance as defined by a reduction of theater time, number of fluoroscopy images taken, postsurgical complication rates, and an increase in pinning accuracy. In all metrics, intervention trainees will be compared with retrospective-matched comparator trainees.

## Methods

### Study Setting

This multicenter, interventional cohort study will involve first-year orthopedic trainees stationed in New Zealand hospitals between 2019 and 2023.

Trainee performance will be collected across hospitals located in Invercargill, Rotorua, Timaru, Palmerston North, Whanganui, Taranaki, Tauranga, Christchurch, Waikato, and Auckland for 2022. The locations of first-year trainees in 2023 are yet to be determined. Retrospective comparator performances will be collected through training logbooks and electronic patient records.

### Eligibility Criteria

The inclusion criterion was being a full-time registered first-year orthopedic trainee in New Zealand between 2019 and 2023.

### Study Design

**Figure 1:**
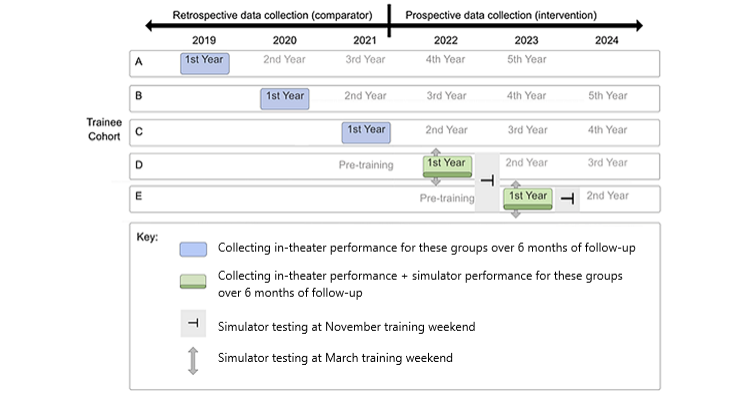
Schematic representation of the intervention and comparator data collection timeline.

The intervention cohort is composed of trainees using the BoneDoc AR simulator at least once per month over a follow-up period of 6 months (May to November) in the years 2022-2023 (Figure 1).

### Recruitment

Intervention group trainees will be approached at training weekends to inform them about the study, to assess eligibility, and to receive consent. Comparator group trainees will be identified from the New Zealand Orthopaedic Association trainee registry and approached via email for this process. Enrollment for comparator group trainees will begin in February 2022, and enrollment for intervention group trainees will occur in the March training weekends of 2022 and 2023.

We aim to include data for 14 trainees in 2022; however, the number of trainees in 2023 is not yet known. The comparator cohort data will be retrospectively audited from trainees who undertook traditional training without exposure to the simulator during their 6 months of follow-up in the years 2019, 2020, and 2021 (n=41). We expect a total sample size of approximately 65 trainees, given an average of 12 new trainees per annum. The comparator group is matched based on their length of registered training at the start of follow-up, that is, first-year trainees with 2 months of registered training.

The in-theater performances of the comparator and intervention groups will be compared using cumulative sum charts for each of the primary outcomes to determine any differences in trends of skill acquisition according to the method of training. The simulator performance of the intervention group will be used to establish a learning curve and compared with in-theater learning curves.

Retrospectively matched trainees have been chosen instead of prospective randomized ones for the comparison group in order to maximize sample size and to avoid contamination. New Zealand only introduces 10-15 trainees per year into the orthopedic training program, and thus a prospective randomized trial will greatly limit the statistical power. There is also the chance of cross-group contamination as there is no way of ensuring comparator trainees located in the same area as intervention trainees will not use the simulator.

### Interventions and Comparators

#### Intervention

The simulator will be first administered at the March training weekends for each year. We will use these training weekends to test participants at baseline on the simulator and collect demographic data via survey. The simulator will be set up as one of the 15-minute assessment stations in the training weekend. Simulator sets will be distributed to individual trainees following the training weekend. Upon delivery of the simulator sets, the principal investigator will also provide aid to the trainee in identifying an appropriate environment for the simulator and address any setup issues.

It is then expected that trainees will undertake, at minimum, monthly 15-minute practices on the simulator for the next 6 months of training. These practice data are sent to a cloud server after each use. Note that the simulator training will be in addition to any routine training they will receive at their respective hospitals. Over this time, primary end points for each in-theater case will be taken over the course of follow-up through District Health Board (DHB) records, including patient picture archiving and communication system (PACS) records and trainees’ logbooks.

We will request intervention trainees to write out relevant patient data and end point results as the cases present to maximize accuracy. Trainees will be reminded monthly by email to provide case data and practice on the simulator. Their simulator performance will be assessed again in a controlled environment during the November training weekend. At this point, secondary end points will also be collected via survey.

#### Comparators

The comparator cohort’s follow-up will begin in February of each year. This is due to the change to the training schedule instated at the end of 2020 whereby trainees began their rotations in February instead of the prior December.

Primary outcomes will be collected retrospectively via trainee logbooks and patient PACS records for retrospective data. Secondary outcomes will not be collected for comparators.

### Data Collection

#### Outcomes

##### Primary End Points—Collected for Each In-Theater Case Over Follow-up

- Theater time and radiation time in seconds (radiation time may not be available from retrospectively assessed notes but will be attained where possible)—collected from procedure reports from electronic patient records
- Number of fluoroscopy shots—collected from patient PACS records
- Pin accuracy—accuracy is defined based on procedural recommendation literature—analyzed from patient PACS records
- Complication/nonideal union incidence—stratified for types of complications (nerve injury, vascular injury, loss of reduction, compartment syndrome, and infection)—collected from reviews of medical records and letters of referral from electronic patient notes

These primary end points have been determined as measurements of trainee performance, which are relatively easily retrieved from patient records and trainee logbooks.

The number of fluoroscopy shots and radiation time can be used to approximate the amount of radiation patients and staff are exposed to. In particular, for patients, theater time and radiation exposure have been shown to correlate to a higher chance of complications and thus should be avoided [21]. Studies have shown a decrease in both measurements for SCHF treatments with increased trainee proficiency [22,23]. Pin accuracy affects the stability of union, and there are well-established guidelines for ideal position of pins.

Ultimately, postoperative complications and nonideal union are the most crucial outcomes the trainees should aim to avoid. We do not expect to see high rates of these for either group, but overall rates will be monitored for comparison.

##### Secondary End Points—Experience With the Simulator and Surgery—Collected at the End of Follow-up Training Weekend for Intervention Trainees 

- Total number of times using the simulator over follow-up—collected from the BoneDoc AR app
- Frequency of using the simulator—collected from the BoneDoc AR app
- Perceived familiarity with SCHF by the end of the follow-up—determined from a 5-point Likert scale administered with a brief electronic survey:
  Unfamiliar
  Somewhat unfamiliar
  Neither familiar nor unfamiliar
  Somewhat familiar
  Familiar
- Total number of SCHF treatments undertaken over follow-up—calculated using the entries from trainee logbook
- Number of mistakes while using the simulator—defined by the number of times the trainee reconsiders pin, collected from the BoneDoc AR app
- Pinning accuracy on simulator—final pin and range of mistakes as calculated by the simulator—collected from the BoneDoc AR app

#### Patient Data

Data such as the National Health Index (NHI) number and surgical details will be collected from trainee logbooks. The following data will be collected from the corresponding DHB records: NHI number; time and date of presentation to the emergency department; age; sex; ethnicity; side of fracture; Gartland classification of fractures—type I, type II, type III, and type IV; the presence of consultant or any other assistants in surgery.

### Statistical Analysis

Operative outcomes using data taken before or without ongoing exposure to the simulator will be compared to outcomes using data from trainees who have used the simulator after 1 year. Comparisons will be performed using Student *t* tests or Wilcoxon Rank Sum tests (depending on the distribution of the data) for theater time and chi-square or Fischer exact tests (depending on the count of the outcome) for the number of complications, number of accurate pin placements, and number of mistakes.

Primary end points will be plotted on separate cumulative sum curves to show trends in performance over the course of follow-up by individuals and aggregated according to pre- and post–1-year simulator training. Control limits will be determined based on values identified in the literature and consultation with senior orthopedic consultants [22-24].

Accuracy of pin placements in subsets of images measured twice must be based on anterior-posterior, lateral, and oblique radiographs. Measurements taken will need to be compared for intra- and interobserver reliability using Bland-Altman plots and intraclass correlation scores.

The results from the familiarity questionnaire will be summarized as responses per category.

Univariable ordinary least squares regression analyses will be used to examine the association of theater time, number of complications (including nonideal unions), and number of x-ray shoots with independent variables. Logistic regression will be used to examine the association of accurate pin placement (yes or no) with independent variables. Independent variables will be the number of times the simulator is used, the number of SCHF procedures performed prior to intervention, Gartland classification, scores on tests of spatial awareness, and the number of SCHF procedures performed during the intervention. Independent variables that are significantly associated with a primary end point (*P*<.05) will be included as covariates in multivariable analyses. Appropriate model diagnostics, and where required, data transformations will be used.

### Software

All analyses will be performed using the statistical analysis software R (R Foundation) [25].

### Data Management

Adverse events are unexpected with the use of the simulator, as trainees have operative experience and there is a low infection risk. Nevertheless, an independent data monitoring committee (DMC) has been setup consisting of 2 clinicians and will be reported to by a study researcher. Any adverse events will be reported immediately to the committee by trainees directly, and the committee may request an interim analysis of the data collected up to that point. The committee can request that the study be paused or discontinued based on the data they receive. The DMC will meet every 6 months to discuss this project.

Study researchers will ensure that the participants’ anonymity is maintained. The participants will be identified only by a participant’s ID number. Individual patients will not be contacted by a researcher. Identifiable patient and participant data will be anonymized and stored on secure University of Otago servers, and will only be accessible by the researchers directly involved with this data collection. Analysis will be performed only on anonymized data sets with an anonymous identifier instead of NHI numbers. The study will comply with the Data Protection Act, which requires all data to be anonymized as soon as it is practical to do so. Data will be stored for 10 years after the conclusion of the study and subsequently destroyed.

### Ethical Considerations

The principal investigator will ensure this that study is conducted in full conformity with the current revision of the Declaration of Helsinki (last amended in October 2000, with additional footnotes added in 2002 and 2004). The principal investigator will ensure that this study is conducted in full conformity with relevant regulations and with the ICH Guidelines for Good Clinical Practice (CPMP/ICH/135/95) July 1996.  Our study was approved by the University of Otago Health Ethics committee (reference HD21/087).

Any important protocol modifications will be communicated in a timely manner to trial participants already enrolled and the DMC in a timely manner.

The results of this study will be disseminated through peer-reviewed publications and conference proceedings. Trial participants will be provided with the option to receive notification of any disseminated results upon enrollment.

## Results

As of February 2022, a total of 8 retrospective comparators have been recruited by email.

## Discussion

### Strengths and Limitations

This study is not a randomized trial and uses historical comparators, which may limit our ability to generalize the results due to confounding factors. We aim to mitigate the effects of potential confounding factors by matching comparators based on training experience. Potential confounding variables will be collected at baseline for both groups and explored for associations with our outcome measures. In monitoring the learning curves of both groups over time, a difference-in-difference analysis will be performed to explore simulator effects. The quality of patient data and logbook entries by historical trainees will determine the quality of our comparator data; however, we are confident that the primary outcomes we have chosen are sufficiently routinely collected for surgical cases.

This is the first surgical simulator validation study conducted across all New Zealand centers with early-stage trainees and will be invaluable for establishing a pathway for future New Zealand studies. In collaborating with the training organization at the early stages of testing, and continuous collaboration over the course of the project, the formal integration of this simulator into training can be streamlined. Collaborations for this study also exist with orthopedic surgeons in the United States. Successful multicenter testing in New Zealand is likely to lead to further testing in the United States, thereby improving the external validity of this simulator and possible integration into international training programs. 

### Conclusions

The results of this pilot study will show the feasibility and efficacy of simulator use within New Zealand’s public health system. The long-term benefits to society will be both for the education of registrars and the health outcomes of patients. In particular, for trainees in localities with lower populations, this simulator can address the disparity in case volumes for those that would otherwise have been deprivileged by their location. Moreover, this simulator has been developed with minimizing costs and increasing accessibility as key objectives; it is cheaper than many currently available models and has low requirements for the environment. We envisage its frequent use as a valuable tool within hospital systems.

## Appendix

Multimedia Appendix 1 Peer-review reports (1) from the University of Otago - Dunedin School of Medicine Research Student Support Committee (New Zealand).

Multimedia Appendix 2 Peer-review reports (2) from the University of Otago - Dunedin School of Medicine Research Student Support Committee (New Zealand).

## Abbreviations

AR: augmented reality
CRPP: closed reduction and percutaneous pinning
DHB: District Health Board
DMC: data monitoring committee
NHI: National Health Index
PACS: picture archiving and communication system
SCHF: supracondylar humeral fractures

## Data Availability

The deidentified data will be attached as a multimedia appendix to the final published manuscript once the study is completed.
