# Supplementary material for: Transfer Validity of Pediatric Supracondylar Humeral Fracture Pin Placement Practice on In-Theater Performance by Orthopedic Trainees Using an Augmented Reality Simulator: Protocol for a Pilot Interventional Cohort Study With a Retrospective Comparator Cohort
Source: JMIR Res Protoc. 2023 Aug 2;12:e38282. doi: 10.2196/38282 (PMC10433022; doi:10.2196/38282)
Supplement: Multimedia Appendix 1 [file resprot_v12i1e38282_app1.pdf]

UNIVERSITY of OTAGO
Te Whare Wānanga o Otāgo
NEW ZEALAND

SCIENTIFIC PEER REVIEW: Reviewer templates

Reviewer Template Version 1

Date: 14 September 2021
Research Title: Transfer validity of Paediatric supracondylar humeral fracture pin placement practice on in-theatre performance by Orthopaedic trainees using an augmented reality simulator
Researcher Name: Dr Phil Blyth

Reviewer Name: Associate Professor Stephanie Woodley, Department of Anatomy
Reviewer signature ____________

Recommendation: Approve / Revise minor / Revise major / Decline

REVIEW GUIDELINE	GUIDELINE PROMPTS	COMMENTS
Relative merit of the research	• Important, worthwhile and justifiable.<br>• Addresses a health issue that is important for health and/or society.<br>• Aims, research questions and hypotheses build on and address gaps in existing knowledge.	This is an interesting, novel study which seeks to explore the effects of incorporating augmented reality technology (BoneDoc simulator) into orthopaedic surgical training. Little research has been conducted in this field, and this project will be the first simulator validation study to be conducted across New Zealand, in collaboration with the New Zealand Orthopaedic Association training scheme. The project is justifiable in terms of assessing the validity of this training approach by addressing three main research objectives. This project will have educational value (for orthopaedic trainees) and in the longer term may translate to enhanced health outcomes for patients (in this case, children with arm fractures) and provide a springboard for strengthening international collaborations.
Design and methods	• Quality of study design<br>• Robustness of the methods used.<br>• Includes a description of sample recruitment and characteristics (including number, gender and ethnicity where relevant) proposed methods of data analysis.<br>• Timelines for the research included	This cohort study has both prospective and retrospective elements, incorporating five cohorts of trainees. It is evident that a lot of work has gone into developing the protocol, but in places this is relatively complex. This may partially be a reflection of the three objectives, which effectively represent three different

Department of Anatomy | School of Biomedical Sciences | University of Otago
Te Tari Kikokiko | Te Kura Mātai Rongoā-Koiora | Te Whare Wānanga o Otāgo

PO Box 56, Dunedin 9054, New Zealand
Tel +64 3 479 7362 • Fax +64 3 479 7254 • Email anatomy@otago.ac.nz
www.otago.ac.nz/anatomy/

		studies. While Figure 1 is a useful guide for the prospective cohorts, I found it difficult to interpret exactly which cohorts were involved/being compared across the different phases of the study, and suggest that this could be clarified. The trainees in the prospective (interventional) group will be assessed for eligibility and consented at the training weekends, however further details as to how this will be undertaken would be useful (including when participants will receive the information about this study). It is unclear how the retrospective (comparator) group will be approached and consented.
Feasibility of the research	• Overall strategy, methodology and analyses are well reasoned and appropriate to achieve the specific aims of the project.<br>• Likely to improve scientific knowledge, concepts, technical capacity or methods in the research field, or of contributing to better treatments, services, health outcomes or preventive interventions.<br>• Achievable within the specified timeframe<br>• Researcher/research team has the appropriate experience and expertise	The timeline for this project has been detailed and it seems feasible that the methods will address the aims of the project. One potential barrier could be that the trainees do not perform the required number of simulator practices, but it is noted that they will receive monthly reminders. The findings of this study will enhance knowledge about the validity of simulator training in an orthopaedic setting (and compare this to "traditional training"), which will have flow-on effects in terms of enhancing surgical skills and patient outcomes. The PI has a medical background in orthopaedics and is well-connected to and supported by orthopaedic and research colleagues with the appropriate experience and expertise.
Presentation of the application	• Appropriate overall presentation, including structure, 'understandability', clarity and readability<br>• In general the way in which the application reads and gets the message across reflects well planned and conceived research.	The protocol is well-organised into sections (the table of contents page is probably not needed). As noted above, clarity could be enhanced in places so to improve the descriptions of the different phases/comparator groups. The information relating to Endpoint Measurements could also be reframed so to clearly specify the primary and secondary outcomes. The section on adverse events needs to stipulate what these relate to in the context of this study as currently they are very generic.
Other comments	Any reviewer observations that are not covered in the points above	Overall this study addresses a novel topic, that will form part of a PhD thesis and provide data which is will be valuable in both national and international contexts. I look forward to following this project and its findings.
