# Supplementary material for: Transfer Validity of Pediatric Supracondylar Humeral Fracture Pin Placement Practice on In-Theater Performance by Orthopedic Trainees Using an Augmented Reality Simulator: Protocol for a Pilot Interventional Cohort Study With a Retrospective Comparator Cohort
Source: JMIR Res Protoc. 2023 Aug 2;12:e38282. doi: 10.2196/38282 (PMC10433022; doi:10.2196/38282)
Supplement: Multimedia Appendix 2 [file resprot_v12i1e38282_app2.pdf]

Date 8/9/21

Research Title____ Transfer validity of Paediatric supracondylar humeral fracture pin placement practice on in-theatre performance by Orthopaedic trainees using an augmented reality simulator

Researcher Name_________Dr Phil Blyth____

Reviewer Name___Dr Steve Gallagher

Reviewer signature ________ ____________________________

Recommendation: Approve / Revise minor / Revise major / Decline

REVIEW GUIDELINE	GUIDELINE PROMPTS	COMMENTS
Relative merit of the research	• Important, worthwhile and justifiable.<br>• Addresses a health issue that is important for health and/or society.<br>• Aims, research questions and hypotheses build on and address gaps in existing knowledge.	• The background, para 2, could make a stronger link between the limited nature of exposure to SCHF during training and its importance a level 4 milestone providing an opportunity for enhanced training opportunities<br>• Are there any references for the extent of the apprenticeship model in orthopaedic training globally? It is referred to as status quo in para 4, but supporting evidence is not supplied. While it might be the experience of the investigators, and I'm sure it is the case globally, it would be good to provide some evidence to support the statement if possible.<br>• In para 4, the authors state "In addition, the simulator can provide objective scores on a standardised model, which are not realistically possible in the real world." This assertion would benefit from further explanation – how does this simulator address this real world shortcoming?<br>• Consider adding a reference to the following in reference to transfer and predictive validity<br>○ Harris DJ, Bird JM, Smart PA, Wilson MR, Vine SJ. A Framework for the Testing and Validation of Simulated Environments in Experimentation and Training. Front Psychol [Internet]. 2020 [cited 2021 Sep 8];0. Available from: https://www.frontiersin.org/articles/10.3389/fpsyg.2020.00605/full<br>• Also worth discussing the shortcomings in literature regarding AR transfer validity (this is a good thing, makes the application worthwhile), eg

DSM Education Unit, Deans Department
Dunedin Hospital, Great King Street, PO Box 56, Dunedin 9054, New Zealand
Tel +64 3 470 9837 • Email dsm@otago.ac.nz
www.otago.ac.nz/healthsciences

		- Barsom EZ, Graafland M, Schijven MP. Systematic review on the effectiveness of augmented reality applications in medical training. Surg Endosc [Internet]. 2016 [cited 2021 Sep 8];30(10):4174–83. Available from: https://www.ncbi.nlm.nih.gov/pmc/articles/PMC5009168/ - I think the objectives could be stated more simply and clearly. As written, they take a bit of effort to understand. For example, - Objective 1: To examine transfer validity of trainee practice on the BoneDoc AR simulator by comparing in-theatre performance of trainees who have used the simulator with retrospectively collected in-theatre performance data from trainees who have not used the simulator. - Vs - Objective 1: Examine the transfer validity of the BoneDoc AR simulator amongst trainees. We will assess the in-theatre performance of trainees who have used the BoneDoc AR simulator and compare this with retrospectively-collected data from a different group of trainees who have not used the BoneDoc AR simulator. - Objective 2 is not clear to me – are you proposing to predict in-theatre performance by using performance on the simulator? This only becomes clear when reading further into the method, and would be preferable to have more clearly articulated in the background section - Overall I think this is an important and worthwhile area of investigation – the gaps in the field could be better established and the objectives more clearly written and explained.
Design and methods	- Quality of study design - Robustness of the methods used. - Includes a description of sample recruitment and characteristics (including number, gender and ethnicity where relevant) proposed methods of data analysis. - Timelines for the research included	- This appears to be generally well designed with appropriate matching of the two cohorts - It is not abundantly clear to me why there is a second “1st year” group assessed in 2023 as depicted in Figure 1. This group doesn’t appear to get follow up so this would seem irrelevant to the question posed in objective 3? I may be missing something. It is proposed in section 4.4.7 that this group would be a comparison group for the first cohort who are at year 2 in 2023, but a between groups comparison doesn’t seem like a good test of training effects – would you not be better to try and predict performance in year two using frequency of engagement with the simulator and/or performance on the simulator as a predictor variable for performance at year 2? - I think this sentence is justifying this group, but the rationale given is not clear to me: “This particular retrospective comparator cohort has been chosen to ensure there is no possible contamination due to accidental exposure of simulator to comparator trainees during their three years of follow-up. - Section 4.4.4 expresses a desire for trainees to engage with the simulator at least monthly. It’s not clear from the protocol how this will be encouraged. For example, is the data on the cloud server monitored and are reminders planned?

		• It's not clear to me why the section on adverse events is included. While it is abundantly clear how important this is more classifying adverse events that occur during surgery, presumably this is part of clinical practice regardless of whether the trainee is involved in the study or not? So is this section intended to state that the study participants will be monitored to identify if they are exposed to any adverse events during the study?
Feasibility of the research	• Overall strategy, methodology and analyses are well reasoned and appropriate to achieve the specific aims of the project.<br>• Likely to improve scientific knowledge, concepts, technical capacity or methods in the research field, or of contributing to better treatments, services, health outcomes or preventive interventions.<br>• Achievable within the specified timeframe<br>• Researcher/research team has the appropriate experience and expertise	• What are the anticipated number of participants? Am I correct is assuming that the number of places available for year 1 trainees in 2022 (14) is the sample size for each year? The analyses proposed seem to be well chosen, but some depend on a certain number of observations in order to be valid, particularly the least-squares regression. I'm sure that the investigators have thought this through but it's not clear to me from reading the proposal.<br>• I note that some data requires direct entry by the participants as cases occur – I wonder if this is a risk for data collection and what steps are in place to remind trainees to do this and/or data checks throughout the study to confirm that the data is of sufficient quality to support final analyses.<br>• It's not clear if there is any allowance for attrition during the longitudinal study and if this has been accounted for in the planned analyses<br>• Because I am not a clinician, I can't accurately predict how logistically challenging this might be, but to me it feels like there are some factors beyond the experimenter's control that may impact on data collection.<br>• I think that if this study is able to collect all the data and gather enough observations for the planned analyses then this has the potential to improve orthopaedic training for an important procedure.
Presentation of the application	• Appropriate overall presentation, including structure, 'understandability', clarity and readability<br>• In general the way in which the application reads and gets the message across reflects well planned and conceived research.	• Well structured but objectives could be stated more clearly.<br>• I consider the application to provide ample evidence of careful planning.
Other comments	Any reviewer observations that are not covered in the points above	Because I am not a clinician, I may be misunderstanding some aspects of the protocol.
